# Determinants of fetomaternal complication of instrumental vaginal delivery among women who gave childbirth in Southern Ethiopia: a facility-based cross-sectional study

**DOI:** 10.1186/s13104-023-06583-w

**Published:** 2023-11-02

**Authors:** Eskinder Israel, Samuel Abayneh, Dawit Utalo, Temesgen Geta, Tamirat Kassaw, Tamirayehu Shonde, Merihun Gebre

**Affiliations:** 1 School of Public Health, College of Health Science and Medicine, Wolaita Sodo University, Wolaita Sodo, Ethiopia; 2 Department of Maternal and Child Health, Gofa Zone Health Department, Sawula, Ethiopia; 3 Departement of Public Health, Consortium Project at Women Empowerment Action with Amref Health Africa, Wolaita Sodo, Ethiopia; 4 School of Nursing, College of Health Science and Medicine, Wolaita Sodo University, Wolaita Sodo, Ethiopia; 5 School of Midwifery, College of Health Science and Medicine, Wolaita Sodo University, Wolaita Sodo, Ethiopia; 6 School of Medicine, College of Health Science and Medicine, Wolaita Sodo University, Wolaita Sodo, Ethiopia; 7 Department of Maternal and Child Health, Southern Ethiopia Regional Health Beurea, Jinka, Ethiopia

**Keywords:** Fetal, Maternal, Complications, Instrumental delivery, Southern Ethiopia

## Abstract

**Background:**

In Ethiopia, one in five instrumental deliveries among women giving birth resulted in an unfavourable outcome. This study aimed to assess the determinants of feto-maternal complications of instrumental delivery in selected public hospitals of Gamo and Gofa zones, Southern Ethiopia.

**Methods:**

An institution-based cross-sectional study was conducted among 399 women attending selected public hospitals in the Gamo and Gofa zones. Data were collected using data extraction tools using a systematic random sampling technique. The collected data was entered into Epi-data version 3.1 and then analyzed using SPSS version 25. Logistic regression analysis was conducted to determine an association.

**Results:**

One hundred eighty-three (45.9%, n = 183/399) instrumental deliveries were found to be complicated. Primigravida women (AOR: 95% CI: 2.21 (1.35, 3.63), infant birth weight (AOR: 95% CI: 2.56 (1.37, 4.77), post-term pregnancy (AOR: 95% CI: 12.77 (2.92, 55.78), and maternal age (AOR: 95% CI: 7.00 (2.16, 22.64) were associated with fetomaternal complications in instrumental delivery among women who gave birth.

**Conclusions and recommendation:**

A high proportion of women developed fetomaternal complications when compared to local studies. Promotion of antenatal care services, increasing women’s education and empowerment as well as working on capacity building of health care professionals through education and training is cost-effective to reduce the occurrence of fetomaternal complications.

**Supplementary Information:**

The online version contains supplementary material available at 10.1186/s13104-023-06583-w.

## Background

Instrumental deliveries are vaginal deliveries conducted with the aid of instruments either by vacuum or forceps to reduce the risk of intrapartum maternal and fetal complications [1, 2]. It is one of the key elements of essential obstetric care and scaling up its use in resource-limited settings such as in Ethiopia has a double advantage [3]. Globally, its ideal rate is often unknown and varies greatly between settings both in developed and developing countries [2, 4]. Feto-maternal complication (FMC) due to instrumental delivery reported in developed countries is minimal because of their advanced skill in obstetric case management and the presence of good obstetric equipment [5, 6]. Sub-Saharan Africa (SSA), where the greatest burden of this complication happens suffers disproportionately and lacks the minimum of this putting both the woman and her infants at an increased risk [7–9]. On average, a 1-3% incidence rate was reported in SSA counties such as Niger, Mali, Burkina Faso, and Nigeria [10].

In Ethiopia, one in five of the instrumental deliveries among women giving birth resulted in an unfavourable outcome [11]. A recent study conducted in Ethiopia indicated that the prevalence of maternal complications in instrumental delivery was 12.1% [11]. Various research conducted in Africa showed that women from rural areas, infant birth weight, being primigravida, poor maternal effort, prolonged labour, multiparty, post-term pregnancy, no antenatal care attendance, and older maternal age were factors associated with FMC in instrumental delivery [8, 11–13].

FMC does indeed not only show maternal and child morbidity and mortality but its effect economically and socially on the entire family and community as a whole [6, 11, 14]. Despite this, few studies were done among women on determinants of FMC of instrumental delivery in Ethiopia. So, this study is aimed at assessing determinants of FMC of instrumental delivery among women who gave birth in selected hospitals of Gamo and Gofa Zone, Southern Ethiopia, 2022.

## Methods and materials

### Study design, period, and setting

A facility-based cross-sectional study design was employed in public hospitals of Gamo and Gofa zone, southern Ethiopia from 01 March to 30 May 2022. Gamo and Gofa are zonal structures found in the South Nation, Nationalities, and Peoples Region (SNNPR), Ethiopia. Gamo Zone is centered at Arbaminch town while Gofa Zone is centered at Sawla town and away 505 and 516 km from Addis Ababa, the capital of Ethiopia respectively. Five public hospitals were found in the Gamo zone (Arbaminch General, Kamba, Geressie, Chencha, and Selamber) and two in the Gofa zone (Sawla General & Laha District Hospital).

### Population

All women who gave birth through the use of the instrument at public hospitals of Gamo and Gofa zones were the source population. All selected women who gave birth through the use of instruments in selected public hospitals of Gamo and Gofa zones during the time of data collection were the study population. All women who gave birth at Gamo and Gofa zone public hospitals were included. Women with incomplete medical records, and who gave birth by cesarean sections were excluded.

### Sample size determination

**Fig. 1:**
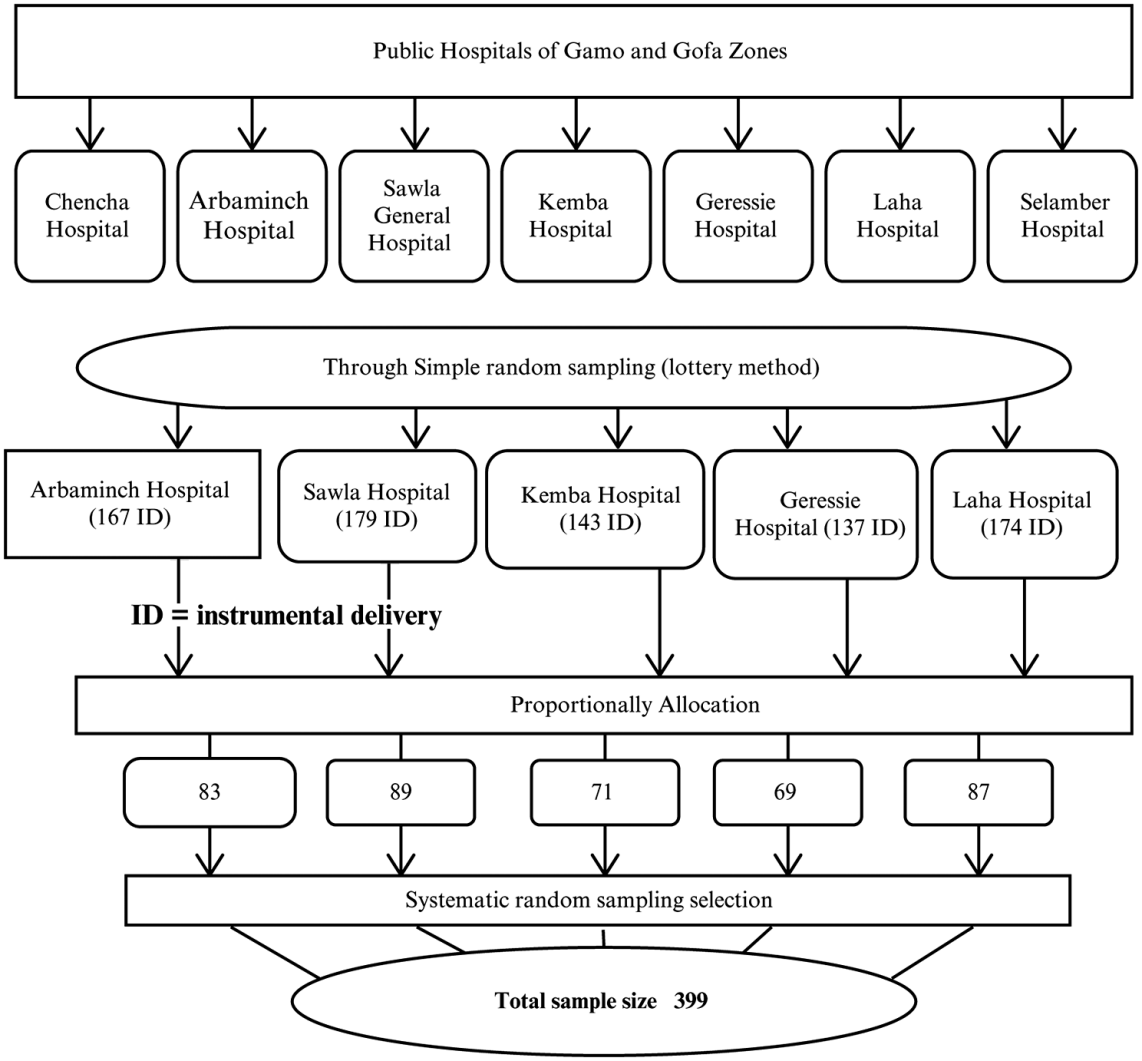
The diagrammatic presentation of the sampling technique on the determinants of feto-maternal complications of instrumental delivery in selected public hospitals of Gamo and Gofa zones, southern Ethiopia, 2022

The sample size was calculated using a single population proportion formula with considering the following assumptions: 95% confidence level, 5% margin of error, 45.4% of the proportion of feto-maternal complications of instrumental deliveries in Suhul General Hospital, Shire, North-West Tigray, Ethiopia [6].

$$\text{n=}\, \frac{\text{Z}^{2}\text{p}\, \text{(1-p)/}}{\text{d}^{2}}$$,

Where n = the required sample size, Z is a confidence interval of 95% = 1.96, the estimated proportion and d is a marginal error and yields a total sample size of 380 and when adding a 5% non-response rate to this, it gives 399 total sample size. The number of instrumental deliveries reported in the previous two months was 801. Firstly, five public hospitals were selected randomly from the total hospitals found in Gamo and Gofa zones. Then the sample size was allocated proportionally to the number of each hospital through a systematic random sampling technique (k = 2) based on their delivery registration (Fig. 1).

### Operational definition

#### Feto-maternal complications

defined as having at least one maternal or fetal complication diagnosed by one of the health care providers [8].

### Data collection tools and procedure

The data extraction tools were modified and adapted from different available related literature. The tools consist of sociodemographic characteristics (age, residence, educational level, occupation, religion), obstetrical characteristics (gravidity, parity, antenatal care attendance, gestational age, fetal presentation, use of vacuum delivery, use of forceps delivery, types of instrumental delivery, birth attended health providers), maternal complications (indication of instrumental delivery, presence of maternal complication, identified postpartum maternal complication), fetal complications (presence of fetal complication, identified fetal complication, APGAR score, infant birth weight). The English version of the data collection tool was initially translated into the Amharic language by independent translators who had well health background and then retranslated back to the English language to check for possible consistency. Data was collected by BSc midwives who trained in BEMONC (Basic Emergency Obstetric and Neonatal Care) training and working in nearby hospitals (wolaita Sodo Univeristy Comprehensive Specialized Hospital) to minimize potential bias. Two days of training were given for data collectors and supervisors in Amharic and English language on how to ask and fill in the questions, and how to approach women by the principal investigator. The pretest was done among 5% (n = 20) of the total sample size at the nearby hospital (Wolaita Sodo University Comprehensive Specialized Hospital) to ensure the appropriateness, simplicity, clarity, understandability, and coherence of the tools before collecting the actual data.

### Data analysis

The collected data was first entered and then cleaned using Epi data version 7.2.2.6, and finally exported to SPSS 25, for further analysis. Bivariate logistic regression analysis was carried out to identify eligible variables for the later analysis with a significance level of p < 0.25. Then, significant variables in the bivariate logistic regression analysis were included in the multivariate logistic regression analyses to identify determinants. Finally, an odds ratio with 95% CI variables at a significance level of p < 0.05 was used to declare the presence of an association between independent variables and FMC.

### Ethical considerations

Ethical clearance was first approved by the Institutional Review Board of Arbaminch University, College of Medicine and Health Sciences (ref. Number: IRB/387/14) and then a support letter was also written to Gamo and Gofa zone hospitals for cooperation. Informed consent was taken after explaining all the necessary information to the woman. The data collected was kept confidential.

## Results

### Socio-demographic characteristics of the woman

A total of 399 (100%) women were involved in the study making the response rate 100%. More than three-fourth, 304 (n = 304/399, 76.2%) women were aged between 20 and 34 years. Nearly one-fourth (n = 96/399, 24.1%) of the woman completed their primary level education, and above half, (222/399, 55.6%) were housewives in their occupation (Table 1).

**Table 1:** Sociodemographic characteristics of the women in selected public hospitals of Gamo and Gofa Zones, Southern Ethiopia, 2021 (n = 399)

Characteristics	Categories	Frequency	Percentage
Age (in years)	< 20 20–35 ≥ 35	72 304 23	18.0 76.2 5.8
Residence	Urban Rural	215 184	53.9 46.1
Educational level	Not able to read and write	98	24.6
	Able to read and write	30	7.5
	Elementary (1–8)	96	24.1
	High school (9–12)	113	28.3
	College and above	62	15.5
Occupation	Housewife Civil servant Merchant Student	222 98 51 28	55.6 24.6 12.8 7.0

### Obstetric characteristics

Over half (n = 234/399, 58.6%) of the women were primigravida. More than half, (215/399, 53.9%) of the woman had no antenatal care attendance. One hundred and seventy-six (176/399, 44.1%) women were attended by integrated emergency surgical officers (IESO) in their childbirth (Table 2).

**Table 2:** Obstetric characteristics of the woman in selected public hospitals of Gamo and Gofa Zones, Southern Ethiopia, 2021 (n = 399)

Characteristics	Categories	Frequency	Percentage
Parity	0	133	33.3
	1	82	20.6
	1–4	159	39.8
	≥ 5	25	6.3
Gravidity	Multigravida	165	41.4
	prim gravid	234	58.6
Antenatal care	Yes	184	46.1
	No	215	53.9
Gestational age	Term	327	82.0
	Preterm	20	5.0
	Post-term	52	13.0
Fetal Position	Occiput anterior	396	99.2
	Occiput transverse	3	0.8
Instrumental delivery type	Outlet	326	81.7
	Low	67	16.8
	Mid	6	1.5
Attended clinician	Emergency surgical officer	176	44.1
	General practitioners	13	3.3
	Health officer	135	33.8
	Midwives	75	18.8

### Fetomaternal conditions of the woman

**Fig. 2:**
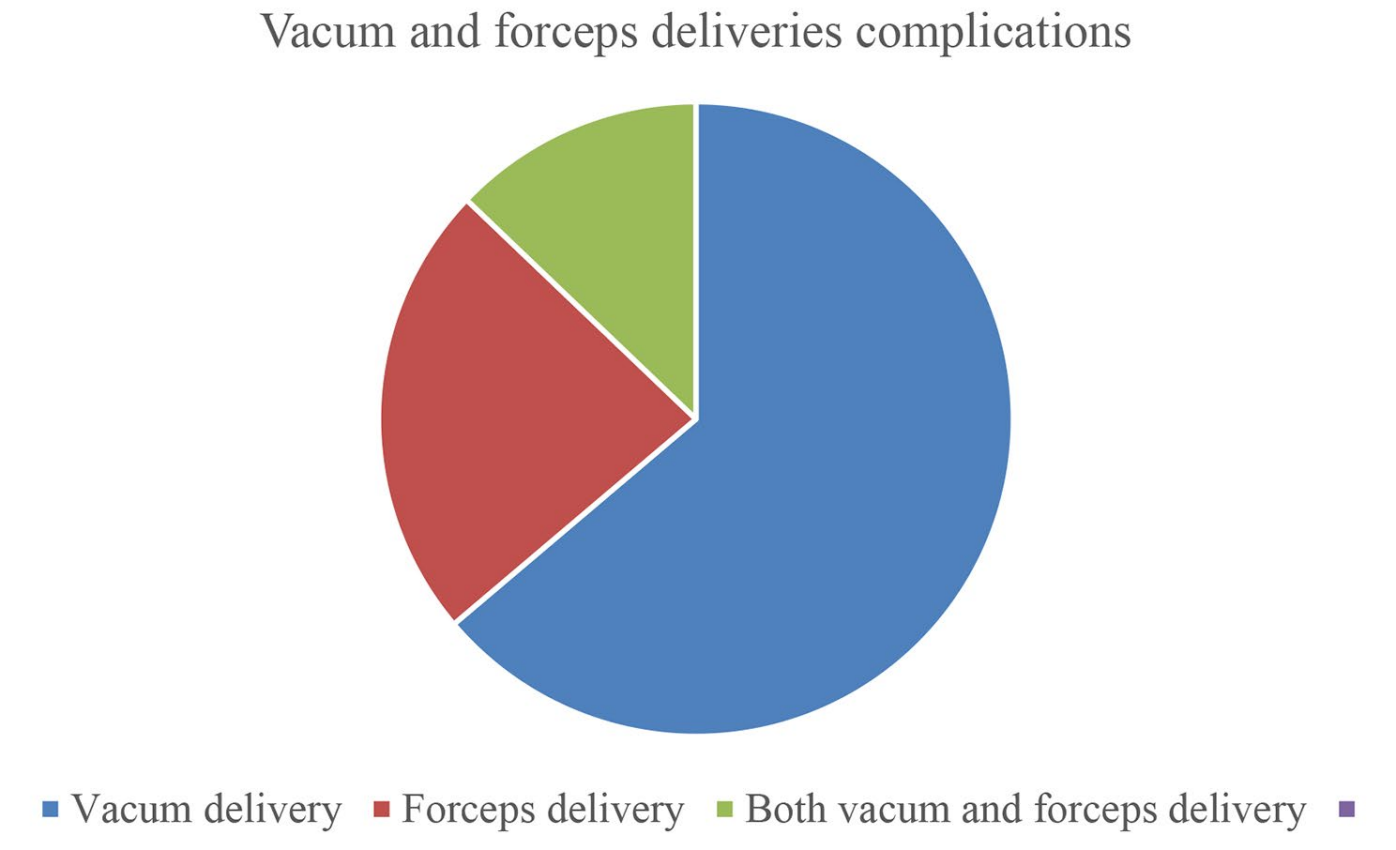
A figure showing vacuum and forceps delivery complications of the women in selected public hospitals of Gamo and Gofa Zones, Southern Ethiopia, 2022

One hundred eighty-three (n = 183 /399, 45.9%) fetomaternal complication was reported in this study. Among those, nearly half, 170 (n = 170 /399, 42.6%) of the complications were maternal (n = 91/399 maternal only and 79/399 with both maternal and fetal), and 92 complications (n = 92/399, 23.0%) were fetal (n = 13/399 fetal only and n = 79/399 with both maternal and fetal complications) (Table 3). In this study, vacuum complications comprise 33.5% (Fig. 2).

**Table 3:** Feto-maternal condition of the woman in selected public hospitals of Gamo and Gofa zones, Southern Ethiopia, 2023 (n = 399)

Characteristics	Categories	Frequency	Percentage
Both fetal and maternal complications	Yes	183	45.9
	No	216	54.1
APGAR Score	0–3	6	1.5
	4–6	143	35.8
	7–10	250	62.7
Total maternal complications	Yes	170	42.6
	No	229	57.4
Birth Weight in (gram)	500–999	24	6.0
	1000–1499	23	5.8
	1500–2499	41	10.3
	2500–3999	226	56.6
	≥ 4000	85	21.3
Total fetal complication	Yes	92	23.1
	No	307	76.9
Indication of Instrumental vaginal deliveries	Fetal distress	184	46.1
	Prolonged 2nd stage	215	53.9

### Determinants of feto-maternal complication of the instrumental Delivery

After controlling the effect of confounders in multivariate logistic regression analysis; primigravida, birth weight, post-term pregnancy, and maternal age were significantly associated with fetomaternal complications of instrumental delivery. Primigravida women were over two times more likely to develop feto-maternal complications when compared with multigravida women (AOR: 95% CI: 2.21 (1.35, 3.63). Infants whose birth weight was > 4000gm had an increased probability of developing feto-maternal complications than their counterparts (AOR: 95% CI: 2.56 (1.37, 4.77). The odds of women with post-term pregnancy had an increased probability of developing feto-maternal complications when compared to women with term pregnancy (AOR: 95% CI: 12.77 (2.92, 55.78). Women aged 20–34 were over seven times more likely to develop feto-maternal complications than women with their counterparts (AOR: 95% CI: 7.00 (2.16, 22.64) (Table S1).

## Discussion

The magnitude of fetomaternal complication found in this study was 45.9% (n = 183/399, 45.9%). Primigravida, birth weight, post-term pregnancy, and maternal age were found to be associated with fetomaternal complications of instrumental delivery in this study. The magnitude found in this study goes in line with the studies conducted in the Swedish population-based study and North-West Tigray that showed similar findings [4, 6]. This similarity could be due to the presence of poor obstetric care, underutilization of health care, and differences in study design. The finding in this study was higher than the studies conducted in the United Kingdom (9.5%) [15], Sweden (8.6%) [4], Uttarakhand state (India) (13.8%) [1], tertiary teaching hospital, India (5.25%) [16], Government tertiary care hospital in Mandya, India (23.18%) [17], Ahmadu Bello University Teaching Hospital Zaria, Nigeria (11.8%) [18], Jimma Medical Center, Ethiopia (4.1%) [13], Felege Hiwot Specialized Hospital, Northwest Ethiopia (12.1%) [19] The possible difference for this could be differences in utilization of maternal health care, hospital set-up differences, the difference in client preferences on the mode of delivery and the difference in the study population. However, lower than the study conducted in Port Harcourt Teaching Hospital in Nigeria (46.3%) and Lumbini Medical College Teaching Hospital, Nepal (17.3%) [20, 21]. This difference might be due to the early women’s presentation to their health facilities, and the presence of good knowledge about the risk factors of PPH.

In the current study, more than half (53.9%) of the woman had not attended their antenatal care service during their facility-based childbirth. This goes in line with the studies conducted in Rwanda (54%) [22]. This is because of similarities in sociodemographic characteristics of the women. But lower than the study conducted in Debretabor town (64.7%) [23] and higher than the studies conducted in Sub-Saharan Africa (41%) [24], Nigeria (42%) [25] and Kenya (48%) [26]. This is mainly due to the difference in obstetric characteristics and variation in the level of health facilities. Various studies indicated that lack of antenatal care follow-up during pregnancy is associated with various adverse fetomaternal complication outcomes including pregnancy-related hypertension, neonatal death, low birth weight, preterm and even death [27–29]. In addition to this, the promotion of women’s empowerment and general education can result in favourable and good maternal health outcomes through increased use of their reproductive rights, freedom for engaging in their treatment outcomes and understanding the prevention modalities including the use of antenatal care service and other maternity continuum care completions thus results in a reduction of fetomaternal adverse outcomes [30–33].

This study also pointed out that primigravida women were over two times more likely to develop feto-maternal complications when compared with multigravida women. This finding is similar to the study conducted in Felege Hiwot Specialized Hospital, Northwest Ethiopia [19], and could be due to the presence of adequate health service utilization by the women in the area. This is because primigravida women often develop late second-stage delay because of rigid perineum and relatively low degree of cephalopelvic disproportion (CPD), and this in turn results in difficulty of instrumental delivery and hence fetomaternal complication could happen [34]. Additionally, infants whose birth weight was > 4000gm had an increased probability of developing feto-maternal complications than their counterparts. These findings are consistent with the studies conducted in India [5], Aksum Saint Mary Hospital, Tigray, Northern Ethiopia [9], and Jimma in 2018 [35]. This could be because women who had an infant birth weight of > 4000gm and had prolonged labor put them at more risk of developing postpartum haemorrhage (PPH) that finally results in uterine atony and perineal lacerations [35].

The odds of women who came with post-term pregnancy had an increased probability of developing feto-maternal complications when compared to women with term pregnancy. This statement goes in line with the studies conducted in Pakistan [8]. This is due to women presented with post-term pregnancy often being at risk for severe respiratory distress, macrosomia, and meconium aspiration syndrome, as well as higher rates of operative vaginal delivery or cesarean delivery, and even stillbirth, which could happen [36].

Additionally, women aged 20–34 were over seven times more likely to develop feto-maternal complications than women with their counterparts. The exact reason for this association remains unclear and needs further research. However, various research reported that women aged beyond 40 are associated with fetomaternal complications due to an increased risk of severe preeclampsia, and gestational diabetes as compared with other age groups [37].

This study has some limitations. Firstly, the study used data from both primary and secondary sources and some information was incomplete, due to either the absence or poor habits of keeping the clinical records of the woman. Secondly, the study did not show any long-term complications for this infant and woman. Despite this, the study tried its best to show the key determinants of FMC of instrumental in the study area.

## Conclusion

This study revealed that a high proportion of women developed FMC when compared with local studies. Promotion of antenatal care services, increasing women’s education and empowerment as well as working on capacity building of health care professionals through education and training is cost-effective to reduce the occurrence of fetomaternal complications.

### Electronic supplementary material

Below is the link to the electronic supplementary material.

Supplementary Material 1

## Data Availability

The dataset used and/or analyzed during the current study is available from the corresponding author upon reasonable request.

## Abbreviations

ANC: Antenatal Care
AOR: Adjusted Odds Ratio
APGAR: Appearance, Pulse, Grimace, Activity, Respiration
CI: Confidence Interval
JUMC: Jimma University Medical Center
NICU: Neonatal intensive care unit
NNR: Non-respondent rate
OA: Occiput anterior position
PPH: Postpartum haemorrhage
SNNPR: South Nation Nationality of Peoples of Region
SPSS: Statistical Package Software for Statistics
USA: United States of America
UK: United Kingdom
