# Supplementary material for: Determinants of fetomaternal complication of instrumental vaginal delivery among women who gave childbirth in Southern Ethiopia: a facility-based cross-sectional study
Source: BMC Res Notes. 2023 Nov 2;16:308. doi: 10.1186/s13104-023-06583-w (PMC10623779; doi:10.1186/s13104-023-06583-w)
Supplement: Supplementary file 1 — Supplementary Material 1 [file 13104_2023_6583_MOESM1_ESM.docx]

**Table S1:** A table showing determinants of fetomaternal complication of instrumental delivery among women who gave childbirth in selected public hospitals of Gamo and Gofa Zones, Southern Ethiopia, 2021 (n=399).

| Variable | | Feto-maternal complication | | COR(95%CI) | AOR(95% CI) |
| --- | --- | --- | --- | --- | --- |
|  |  | **Yes (%)** | **No (%)** |  |  |
| Maternal age | < 20  20-34  ≥ 35 | 59 (81.9)  114 (37.5)  10 (43.5) | 13 (18.1)  190 (62.5)  13 (56.5) | 1  7.5 (3.9, 14.4)  5.9 (2.12, 16.36) | 1  7.0 (3.4, 14.3)**  7.0 (2.1, 22.6) |
| Gestational Age | Term  Preterm  Post-term | 127 (38.8)  8 (40.0)  48 (92.3) | 200 (61.2)  12 (60.0)  4 (7.7) | 1  18.8 (6.6, 53.6)  18.0 (4.6, 69.6) | 1  12.4 (4.1, 37.9)**  12.7 (2.9, 55.7)** |
| Birth Weight (in grams) | 500-999  1000-1499  1500-2499  2500-3999  >4000 | 9 (37.5)  12 (52.2)  19 (46.3)  86 (38.1)  57 (67.1) | 15 (62.5)  11 (47.8)  22 (53.7)  140 (61.9)  28 (32.9) | 1  0.5 (0.1, 1.7)  0.6 (0.2, 1.9)  0.9 (0.4, 2.3)  0.2 (0.1, 0.7) | 1  2.7 (0.9,8.2)  2.1 (0.7, 6.8)  1.8 (0.7, 4.5)  2.5 (1.3, 4.7)** |
| Number of pregnancy | Multigravida  Primigravida | 102 (61.8)  81 (34.6) | 63 (38.2)  153 (65.4) | 1  3.0 (2.0, 4.6) | 1  2.2 (1.3, 3.6)** |

**=p –value <0.05, COR=Crude odds ratio and AOR=Adjusted odds ratio.
